# Policies to Encourage the Use of Biosimilars in European Countries and Their Potential Impact on Pharmaceutical Expenditure

**DOI:** 10.3389/fphar.2021.625296

**Published:** 2021-06-25

**Authors:** Sabine Vogler, Peter Schneider, Martin Zuba, Reinhard Busse, Dimitra Panteli

**Affiliations:** ^1^ WHO Collaborating Centre for Pharmaceutical Pricing and Reimbursement Policies, Department of Pharmacoeconomics, Austrian National Public Health Institute (Gesundheit Österreich GmbH/GÖG), Vienna, Austria; ^2^ Department of Health Economics and Health Systems Analysis, Austrian National Public Health Institute (Gesundheit Österreich GmbH/GÖG), Vienna, Austria; ^3^ Department of Health Care Management, Technische Universität Berlin, Berlin, Germany

**Keywords:** biological, biosimilar, savings, substitution, cross-country comparison, policy measure

## Abstract

**Introduction:** Biosimilar medicines are considered promising alternatives to new biologicals with high price tags. The extent of savings resulting from biosimilar use depends on their price and uptake, which are largely shaped by pricing, reimbursement, and demand-side policies. This article informs about different policy measures employed by European countries to design the biologicals market and explores potential savings from the increased use of biosimilar medicines in Germany.

**Methods:** Policy measures that target the price and uptake of biosimilar medicines were identified based on a prefilled questionnaire survey with public authorities in 16 European countries, who were the members of the Pharmaceutical Pricing and Reimbursement Information network (July 2020). Potential savings that could have been generated in Germany if different measures identified in the surveyed countries had been implemented were calculated for six publicly funded biological molecules. Price data of the Pharma Price Information service and German consumption data for 2018 were used for the calculation of five scenarios.

**Results:** Several countries use a price link policy, setting the biosimilar price as a percentage of the price of the reference biological. Also lowering the price of the reference biological upon market entry of a biosimilar is less frequently used. While tendering of biosimilar medicines in the inpatient setting is the norm, it is rarely employed for biosimilars in outpatient use. Reference price systems and INN prescribing of medicines are the commonly used policy measures in the off-patent market, but some countries define exemptions for biologicals. Substituting biosimilars at the pharmacy level is rather an exception. Potential savings in Germany ranged from 5% (simple price link) to 55% (prices at the level of other countries) for the six studied molecules.

**Conclusion:** Despite some differences, there are discernible tendencies across European countries with regard to their applications of certain policy measures targeting the price and uptake of biosimilar medicines. The potential for savings of some of these policies was clearly demonstrated. Monitoring and evaluation of these rather recent measures is key for obtaining a more comprehensive picture of their impact.

## Introduction

Payers have been concerned with discussions on high-priced medicines for years, regardless of their countries’ ability to pay; these discussions have been fueled by the perception that the expenditures on a number of very expensive medicines threaten the financial sustainability of publicly funded health systems. In some countries, new high-priced, on-patent medicines have contributed to increasing pharmaceutical expenditures in recent years (de Bruijn et al., 2016; Pauwels et al., 2017; Godman et al., 2018; Babar et al., 2019).

Biologicals (i.e. biological medicines) are widely used in the treatment of chronic conditions, such as diabetes and autoimmune disorders, as well as cancers, and they often carry high price tags. By offering more affordable alternatives, biosimilar medicines are considered a promising solution to very high-priced biologicals (Kawalec et al., 2017; Abbott et al., 2019). A biosimilar is a “biological medicine highly similar to another biological medicine already approved (…), called 'reference medicine', in terms of structure, biological activity and efficacy, safety and immunogenicity profile (…)” (European Medicines Agency, 2020a). In the European regulatory framework, most biologicals must be approved centrally for market entry in the European Union (EU). As of August 2020, the European Medicines Agency, which evaluates biosimilars, has approved a total of 72 entities since 2006 (European Medicines Agency, 2020b) compared to 28 biosimilars approved by the US Food and Drug Administration (FDA) (U.S. Food and Drug Administration, 2020).

However, in contrast to marketing authorization, pricing and reimbursement as well as any measures to steer the use of medicines (including enhancing the uptake of generics and biosimilars) are national competences of the EU Member States. The respective policies lead to differences both in the prices and the extent of use of biosimilars across countries (Kanters et al., 2017; Kawalec et al., 2017; Moorkens et al., 2017; Manova et al., 2018; Ferrario et al., 2020; Kim et al., 2020), both of which are important contributing factors to the size of achievable savings from biosimilars (Farfan-Portet et al., 2014; Mestre-Ferrandiz et al., 2016). For Europe, such savings were estimated to range between €11.8 and 33.4 billion for the period 2007–2020, resulting in particular from monoclonal antibodies (Haustein et al., 2012). The market share of biosimilars varies across countries; it remains low, at least for certain substances in some systems (Troein et al., 2019).

In recent years, the savings potential for public budget resulting from biosimilar use has been shown: Tendering for infliximab in Norway in 2015 led to the procurement of biosimilar remsima at a price 72% below the official list price of the reference medicine (GABi Online, 2015); biosimilar infliximab also achieved faster market penetration compared to other biosimilars in Norway (Mack, 2015). Tendering for biologics in Italy also led to lower prices and therefore savings for payers due to competition (Curto et al., 2014). In addition, considerable reductions were observed in the prices of reference medicines (biologicals) upon biosimilar market entry in some countries, as marketing authorization holders of reference medicines have increasingly been applying a price-competition strategy, lowering their prices to make biosimilar substitution unattractive, and thus winning tenders (Troein et al., 2019).

In addition to the effect of pricing and procurement, demand-side measures also play a significant role, inter alia by increasing knowledge about biosimilar medicines, fostering trust and therefore the tendency to use them among patients, prescribers, and dispensers (Mestre-Ferrandiz et al., 2016). One such demand-side measure is substitution at the pharmacy, either of the reference medicine by a biosimilar medicine, or of a biosimilar medicine by a different biosimilar medicine. Another is switching by the doctor, whereby a patient previously treated by one biologic medicine is prescribed a different one, ideally following shared decision-making (Kay et al., 2018).

While the saving potentials of biosimilar use have been established by a number of example cases, an analysis of achievable savings from biosimilar use as a result of specific policy options is lacking.

This article has two aims: on the one hand, to provide an updated overview of the different policy measures employed by governments in European countries to shape the biologicals market; and on the other hand, to explore the potential savings resulting from the use of biosimilar medicines under different scenarios of such measures, taking the German pharmaceutical market as an example.

## Materials and Methods

### Survey on Country-Level Policies

To obtain detailed information on measures regarding price-setting and procurement mechanisms for biosimilars (and generics as comparators) and demand-side measures targeting prescribing doctors and dispensing pharmacists, a survey was carried out among representatives of public authorities responsible for medicines policies in the countries of the study.

Sixteen European countries were selected in this study. They include Austria (AT), Belgium (BE), the Czech Republic (CZ), Denmark (DK), Finland (FI), France (FR), Germany (DE), Ireland (IE), Italy (IT), the Netherlands (NL), Norway (NO), Portugal (PT), Slovakia (SK), Spain (ES), Sweden (SE), and the United Kingdom (UK; described measures mainly represent the situation in England). The study was done from a German perspective, and all other countries except Norway are used as a secondary criterion during price negotiations between payers and manufacturers in Germany. Norway was included because it is known for generating savings from biosimilar medicines (GABi Online, 2015; Mack, 2015; Moorkens et al., 2017). The sample enables insights into different types of health systems in terms of financing and level of organization and covers a range of high-income countries. The study adopts a health system perspective: by focusing on determining potential savings for the public payers, this work aims to explore the contribution of biosimilar policies to financial sustainability and improved access to medicines for patients.

In July 2020, the members of the Pharmaceutical Pricing and Reimbursement Information (PPRI) network were contacted and asked to validate prefilled information on policies applied for biosimilars and generics as of 2020 and to provide missing information. The PPRI network is operated by the Austrian National Public Health Institute (Gesundheit Österreich GmbH, GÖG) and comprises competent authorities for pricing and reimbursement of medicines in 51, mainly European, countries (GÖG, 2020). The authors drafted the preliminary compilation of relevant policies in the countries of the survey based on results of earlier surveys conducted with the members of the PPRI network who are committed to information sharing and used to respond to such queries (Vogler et al., 2014) and, in some cases, evidence reported in the literature. In July and August 2020, the reminders were sent, and a few country representatives were individually contacted for clarification of ambiguous answers. In some countries, information was supplemented by literature, including sources provided by the respondents.

### Calculation of Impact on Public Pharmaceutical Expenditure in Germany

We examined potential savings for the German publicly funded market in 2018 that could have been achieved if different measures identified in the surveyed countries and targeting a) biosimilar prices and b) biosimilar uptake had been implemented and/or prices as observed in other countries were in place, using a sample of six biological active substances.

We determined pharmaceutical expenditure for the medicines included in the sample, based on price data as of December 2018 and volume data from the German publicly funded market (only outpatient setting) for the year 2018. We calculated the expenditure for the different scenarios and compared the expenditure data of each hypothetical scenario to the baseline pharmaceutical expenditure to derive the savings potential.

#### Product Sample

The sample included biologics for which the patent had expired and biosimilars have been brought to the markets of the study countries, as price data of biosimilars in other countries were needed for the calculation in some scenarios. For the selected active substances (adalimumab, etanercept, infliximab, pegfilgrastim, rituximab, trastuzumab), a total of 20 medicines (i.e. in different strengths, pharmaceutical forms, and primary packaging types) were included in the sample (see Supplementary Material 1).

#### Data Sources

The prices of the included products were obtained from the Pharma Price Information (PPI) service of GÖG, which provides price data of official sources in European countries. Ex-factory prices in Euro were used in the calculations, with conversion of data for non-Euro countries (based on the average exchange rate for November 2018 as published by the European Central Bank). For countries where the ex-factory prices were not available due to their policy framework (price setting at the wholesale price level), the PPI service provided ex-factory price data based on statutory wholesale prices and average wholesale margins. The German price data considered the mandatory manufacturer discount of 7% gross on the ex-factory price.

Competent authorities filled out historical data gaps upon request where needed and possible. Out of the sixteen countries in the study sample, prices from Ireland and Portugal were not included in the scenario calculations (for Ireland, only limited data were available; in Portugal, the selected medicines are largely used in hospitals, for which no price information was available).

Consumption data for the included medicines (expressed as items dispensed) in the German publicly funded health system for 2018 were obtained from the AOK Research Institute (Wissenschaftliches Institut der AOK–WidO).

#### Assumptions

Savings can be achieved by changes in price, structure, or volume of prescribed medicines [see also Panteli et al. (2016)]. This work focuses on the first two elements; more specifically, the savings potential for the German reimbursement market in 2018 was calculated for five hypothetical scenarios.

Table 1 summarizes the underlying assumptions of these five scenarios. In two scenarios, a price link policy is assumed: only for biosimilars (scenario 2), and supplemented by a price reduction for the reference biological (scenario 3). The inclusion of all the studied biological substances in the German reference price system is assumed in two further scenarios (scenarios 4 and 5). Scenarios 1 and 5 take prices as observed in other countries, while the other scenarios use German prices.

**TABLE 1:** Selected scenarios and assumptions.

Scen	Name	Assumptions
1	European prices	The German price would be substituted by the lowest observed price of that medicine among the surveyed countries (same active substance, strength and pharmaceutical form and adjusted for pack size)
2	Price link—biosimilars only (short: price link—biosimilars)	A price-link mechanism for biosimilar medicines would be applied in Germany
		It is assumed that at the time of their entry into the German market, all biosimilar medicines would be subject to a 30% price cut in comparison with the price of the reference biological, while the price of the latter would remain unchanged
3	Price link—biosimilars and reference biological (short: price link—all)	Building on scenario 2, a price-link mechanism for biosimilar medicines in Germany would also be applied in this scenario
		A price cut of 15% for the reference biological at the market entry of the first biosimilar medicine in Germany is assumed, adding to the 30% price cut for the biosimilar medicines
4	Reference price system—German prices (short: RPS—DE)	All the studied biological substances would be included in a reference price system in Germany
		The specifications of the existing reference price system in Germany (“Festbetragssystem”) are considered: Medicines of the same active substance, strength, pharmaceutical form and of comparable pack size are grouped into the same cluster, and the reference price (“Festbetrag”) per cluster is calculated based on the highest price of the lower third of medicines in the cluster
		Only German prices are used to calculate potential savings in this scenario
5	Reference price system—European prices (short: RPS—Europe)	Building on scenario 4, a reference price system would be again applied in Germany in this scenario
		The calculation to determine the reference price is repeated, but in this scenario the prices from all the surveyed countries are considered

Scen. = scenario.

Details on the methodology, including a step-by-step description of the calculations for each scenario, are provided in the Supplementary Material 2.

Importantly, the calculations are meant to be illustrative, using Germany as an example to explore possible savings, and are not designed as a budget impact assessment. Given the novelty of several policy measures as well as the rather recent patent expiry for some biologicals (and consequently short period of market availability for some biosimilars), a budget impact assessment in accordance with the established methodological principles would not have been possible for all the policies surveyed and medicines analyzed in this study, and such an evaluation was not intended.

## Results

### Overview of Pricing and Procurement Mechanisms and Demand-Side Measures to Endorse the Use of Biosimilars in European Countries

The following subsections provide an overview of supply and demand-side policies that pricing and reimbursement authorities can use to set the price and increase market penetration of biosimilars in the sixteen studied countries as of July 2020. Supply-side policies concern pricing, procurement, and reimbursement mechanisms, while demand-side measures are targeted at health professionals. These policy measures and their design have been laid down in national legislation.

#### Supply-Side Measures

##### Price Link Policies

When a new generic or biosimilar medicine is brought on the market and/or included into reimbursement, pricing authorities can set its price in relation to the price of the originator or reference medicine if the so-called price link policy has been set out in national legislation: in such cases, the price of a generic or biosimilar medicine has to be set at a certain percentage lower than the originator or reference medicine price. In some countries, price link regulations are also in place for the prices of subsequent generics or biosimilars, which are set based on the prices of generics or biosimilars already on the market. Sometimes, price link policies also include provisions that influence (i.e. reduce) the price of the originator or reference medicine upon generic or biosimilar market entry (PPRI, 2020).

Table 2 provides an overview of the implementation of price link policies for biosimilars compared to those for generics as set out in legislation in the sixteen studied countries, including information on the extent of mandated price difference, where applicable. Several countries use such mechanisms to determine the price of generics, but they do not necessarily apply them to biosimilars as well. In all the studied countries with price link provisions for both biosimilars and generics, smaller price differences are required from the former, i.e. countries allow biologicals to maintain higher prices also in the off-patent market. Few countries in the sample (Germany, Sweden, United Kingdom) do not consider the prices of other medicines to determine generic or biosimilar medicine prices; thus, they do not apply a “price link policy.”

**TABLE 2:** Price-link policies for biosimilar medicines compared to generics as provided in national legislation.

Country	Price link for biosimilars		Price link for generics	
	Applied	Extent of price reduction	Applied	Extent of price reduction
AT	Yes	1st biosimilar: min. −38% of reference medicine	Yes	1st generic: min. −50% of originator
		2nd biosimilar: min. −15% of 1st biosimilar		2nd generic: min. −18% of 1st generic
		3rd and subsequent biosimilars: min. −10% of the previous biosimilar		3rd and subsequent generics: min. −15% of previous generics
		Reference medicine must reduce its price by 30% three months after the 1st biosimilar is included in the reimbursement list		Originator must reduce its price by 30% three months after the 1st generic is included in the reimbursement list
BE	Yes	Biosimilar: −20% of reference medicine	Yes	Generics in category A (essential medicines): − 51,52% of originator
		Further price reductions 12 years after inclusion in the reimbursement list, depending on the market share of the active substance		Generics in category B (all other medicines): − 43,64% of originator
				Further price reductions 12 years after inclusion in the reimbursement list, depending on the market share of the active substance
CZ	Yes	1st biosimilar: −30% of reference medicine	Yes	1st generic: −40% of originator
DE	No	*Not applicable*	No	*Not applicable*
DK	No	No, no price regulation (prices are based on competition that results from processes with tendering elements)	No	No, no price regulation (prices are based on competition that results from processes with tendering elements)
ES	Yes	Biosimilar: −30% of reference medicine	Yes	Generic: −40% of originator
				Upon inclusion into a reference group of the reference price system, reduction of the originator price to the price level of the generics
FI	Yes	1st biosimilar: −30% of reference medicine	Yes	1st generic: −50% or −40% (in cases of new equipment) of originator
FR	Yes	*Outpatient*	Yes	*Outpatient*
		Upon market entry of biosimilars: Biosimilar: −40% of reference medicine and reduction of the reference medicine price by −20%		Upon generic market entry: generic: −60% of originator and reduction of originator price by −20%
		After 18 and 24 months further price reductions, extent (5%, 10% and 15%) dependent on market share (<40%, 40–60%, >60%)		After 18 months price reductions by −7% for generics and −12.5% for originators
		*Inpatient*		*Inpatient*
		Biosimilars: −30% of reference medicine and reduction of the reference medicine price by −30%		Generics: −40% of originator and reduction of originator price by −40%
IE	Yes	Biosimilar: −40% of reference medicine	Yes	Generic: −60% of originator
IT	Yes	Biosimilar:−20% of reference medicine	Yes	Generic: −20% of originator
NL	No	However, the price of biosimilars must be below that of the reference medicine	No	However, the price of generics must be below that of the originator
NO	Yes	Biosimilars may be priced at the same price of the reference medicine, but the prices of biosimilars as well as of reference medicines are subject to cuts upon patent expiry and as well as 6 and 12 months after patent expiry (extent of price cut dependent on the sales) = so-called Trinnpris model (stepped price system)	Yes	Generics: In principle the price must not exceed the originator’s price; price reductions for generics and originators at patent expiry as well as 6 and 12 months after patent expiry (as part of the “Trinnpris” model)
		For active substances not in the “Trinnpris” model: Determination of biosimilar price based on internal and external reference pricing (lowest price)		
PT	Yes	*Reimbursed medicines, outpatient and inpatient setting*	Yes	*Outpatient sector*
		Biosimilars: −20% or −30% (for biosimilars with a market share per substance of more than 5%) of the reference medicine		Generics: −50% of originator and −25% of other generics, when the ex-factory price is <€10 for all packages
		*Non-reimbursed medicines*: No price link		5th and further generics: each new generic −5% of the previous generic, with a threshold of 20% of the originator
				Further generics in the internal reference pricing system: −5% of the lowest-priced medicine in the reference group (minimum market share of 5%)
SE	No	*Not applicable*	No	*Not applicable*
SK	Yes	Biosimilar: −25% of reference medicine	Yes	Generic: −45% of originator
UK	No	*Not applicable*	No	*Not applicable*

##### Tendering

In all the studied countries, tendering is used to procure biosimilars for the inpatient sector (see Table 3). In most countries, procurement is organized at the hospital level, although purchasing networks and collaborations can be in place (e.g. “procurement pools” of university hospitals in Finland). In France, while procurement is usually done by the hospitals, they are incentivized to perform it at regional level. In Denmark, Norway, Portugal, and the United Kingdom, there is centralized procurement of all or some medicines, including biologicals, for the inpatient sector; dedicated procurement agencies to serve public hospitals have been established in the first three countries. Even in countries using price link policies, (biological) medicines are procured via tendering, as procuring institutions hope to achieve lower prices as a result of competition.

**TABLE 3:** Role of tendering and internal reference pricing for biosimilar medicines.

Country	Inpatient sector		Outpatient sector	
	Tendering	Organization	Tendering or tendering elements	Biosimilars in the reference price system
AT	Yes	At hospital level	No	No reference price system
BE	Yes	At hospital level	No	No
CZ	Yes	At hospital level	Yes	Yes
DE	Yes	At hospital level	Yes, as part of discount contracts	Yes (few biosimilars included)
DK	Yes	Centrally (procurement agency *AMGROS*)	Yes, every two weeks	Yes
ES	Yes	At hospital level	No	Yes
FI	Yes	Common “procurement pools” of university hospitals	No	No
FR	Yes	At hospital level or regionally	No	No
IE	Yes	At hospital level	No	No
IT	Yes	Regionally	No	Reference price system in place but biosimilars are not included in these “transparency lists” of equivalent medicines which would imply automatic biosimilar substitution
NL	Yes	At hospital level or by groups of hospital or in collaboration with payers (insurers)	Yes	Yes
NO	Yes	Centrally (procurement agency *Sykehusinnkjop*)	No	Yes
PT	Yes	Centrally (procurement agency *Serviços Partilhados do Ministério da Saúde/SPMS*)	No	No
SE	Yes	Regionally, collaboration of regions	No (not for biosimilars, tendering-like process of defining a “product of the month” only applied for generics)	No reference price system
SK	Yes	At hospital level	Yes	Yes
UK	Yes	Centrally (NHS England)	No	No reference price system; however, country-wide reference/reimbursement prices for Adalimumab

A number of countries also use tendering (or tendering-like elements) to procure off-patent medicines, mostly generics, in the outpatient setting (Dylst et al., 2011; Vogler et al., 2019). A well-known example is the “preference price policy” employed in the Netherlands, wherein health insurers launch a call for tender for active substances and the best bidder will be awarded. For the duration of the contract (usually one year), only this so-called preferred product is reimbursed and pharmacies have to dispense it. Patients who insist on getting dispensed a different product (of the same active substance than the “preferred medicine”) have to pay the difference out of pocket. The “preference price policy” was initially introduced in 2005 as a central instrument (i.e. joint tenders by all Dutch insurers), but a court ruling on competition between insurers in 2008 mandated that insurers have to tender separately. As a result, the range of preferred medicines varies across insurers. Some insurers have been including biosimilars in their preferred medicines schemes (Vogler et al., 2017).

In the Danish outpatient sector, procurement for all reimbursable medicines including biosimilars is based on a tender-like model. Every two weeks, manufacturers have to report to the Danish Medicines Agency planned prices of all their products used in the outpatient sector. The lowest-priced products per substance are considered first choice and covered by the publicly funded health system for the period of the next two weeks. To ensure the availability of medicines, products with the second- and third-lowest price can be dispensed (and be also reimbursed) in case of shortages. Manufacturers who cannot supply are removed from the price list for the tendering time period. Reporting and updating processes are supported by an IT system, which undertakes up to 1,500 updates per two-week period. To mitigate logistic challenges for community pharmacies, cooperation agreements exist with wholesalers and manufacturers, for instance regarding the return of non-dispensed products at the end of the two-week period (Vogler et al., 2017).

In Germany, discount contracts between individual payers (sickness funds) and manufacturers can be considered some sort of tendering for outpatient off-patent medicines. In return for granting discounts on their products, manufacturers can benefit from exclusive dispensing of their medicines. For biologic medicines, the variant of so-called open house contracts has been commonly used: a sickness fund offers to all competing suppliers of a substance a contract that prespecifies the discount rate. Any manufacturer of biosimilar or reference medicines willing to grant this discount rate can join the contract, without conducting any individual negotiations (AG Probiosimilars, 2017).

##### Reference Price System

Most of the countries included in the study have a reference price system in place, which groups products of the same active substance or therapeutically interchangeable medicines into clusters and defines a maximum reimbursement amount per cluster (so-called reference price), thus indirectly regulating medicine prices (Panteli et al., 2016).

Reference price systems are in place in thirteen of the sixteen studied countries, and they vary in methodological terms, regarding how a) the clusters and b) the price benchmarks are determined. This also applies to biosimilars: some countries include biosimilars in the reference price system (e.g. Germany, the Netherlands, Norway, and Spain) and others do not (see Table 3). Since 2009, biologicals can be included in the German reference price system. For instance, infliximab was included in 2016 (G-BA, 2017). In England, even though there is no reference price system, country-wide reference prices for adalimumab 20 and 40 mg were determined in 2019 as the reimbursement price for the NHS (NHS England, 2019).

#### Demand-Side Measures

##### Physicians: Prescribing Requirements and Switching Guidelines

One of the main policies that affects how physicians prescribe medicines is prescribing by International Non-proprietary Name (INN) instead of the trade name of the medicine. INN prescribing has been implemented in many countries, including most of this study (all except Austria, Denmark, and Sweden), and some even mandate it (Table 4). While most studied countries in which INN prescribing is in place have no specific provisions for biosimilars, some explicitly exclude biologicals (e.g. United Kingdom) or recommend against it (Belgium). In Germany and France, in case of a biological INN prescription, pharmacists have to consult with the prescribing doctor before dispensing.

**TABLE 4:** Measures to foster biosimilar prescribing aimed at physicians.

Country	INN prescribing		Prescribing of biologics/biosimilars		
	Applied	Bindingness	Prescribing guidelines and recommendations	Position papers/documents	URL of the position papers/documents
AT	No	Not allowed	Yes; physicians must prescribe the most economical medicine among therapeutically equivalent alternatives (including biosimilars)	“Guidelines for the economical prescribing of medicines and therapeutic aids (RöV 2005)” of the Austrian Social Insurance	https://www.ris.bka.gv.at/Dokumente/Avsv/AVSV_2005_0005/AVSV_2005_0005.pdfsig
BE	Yes	Generally voluntary, but not recommended for biologics	Yes; prescribing quotas (differing per medical specialty) for “cheap medicines” (including biosimilars)	Agreement between the state, some professional associations, the association of hospital pharmacists and the pharmaceutical industry to foster the use of biosimilars, reached in 2016; the agreement became part of the framework agreement with the pharmaceutical industry (“Pact for the future”)	https://www.inami.fgov.be/SiteCollectionDocuments/convention_medicaments_biosimilaires_belgique.pdf
			Recommendation of biosimilar prescribing for naive patients, switching to and between biosimilars is possible (but must be monitored)	2020: Establishment of a task-force to enhance the market dynamics	https://pharma.be/fr/component/attachments/attachments.html?task=attachment&id=235
CZ	Yes	Voluntary	Yes; recommendation of biosimilar prescribing for naive patients, switching to and between biosimilars is possible	Guidelines of the Medical Profession	https://www.linkos.cz/ceska-onkologicka-spolecnost-cls-jep/stanoviska-cos/tiskove-centrum/opinion-of-the-czech-society-for-oncology-on-the-possibility-of-biosimilar-subst/
DE	Yes	Voluntary, but pharmacist has to consult with prescribing doctor in case of an INN prescribing for a biologic (except for “bioidenticals”^a^)	Yes; medicines agreements between prescribers and sickness funds on prescribing quotas and selective contracts (integrated contracts), switching recommended in conjunction with continuous monitoring	Guideline of the Pharmaceutical Commission of the German Medical Profession Association on biosimilars	https://www.akdae.de/Arzneimitteltherapie/LF/PDF/Biosimilars.pdf
DK	No	Not allowed	Yes; recommendation of biosimilar prescribing for naive patients, and of switching to and between biosimilars	Recommendations of the Danish Health Council	https://medicinraadet.dk/anbefalinger-og-vejledninger/vurderinger-af-biosimilaere-laegemidler
ES	Yes	Mandatory	Yes; switching is possible and the decision is taken by the physician	—	—
FI	Yes	Voluntary	Yes; obligation to prescribe the most economical therapeutic alternative for all (not only naive) patients, when biosimilars are available. Prescribing of a more expensive alternative must be justified in writing in the patient’s medical record	Decree of the Ministry for Social Affairs and Health (on prescribing of economical therapeutic alternatives); position paper of the regulatory authority on the interchangeability of reference medicines and biosimilars	https://www.fimea.fi/documents/542809/838272/29197_Biosimilaarien_vaihtokelpoisuus_EN.pdf
FR	Yes	Mandatory, but not allowed for biologics (consulting of pharmacist with prescriber is required)	Yes; contractual obligation for physicians affiliated with social security to prescribe a min of 20% biosimilars for insuline glargine; switching recommended by the National Health Authority	Written recommendation by the National Health Authority	https://www.has-sante.fr/portail/upload/docs/application/pdf/2017-11/bum_medicaments_biosimilaires_v1.pdf
IE	Yes	Voluntary	Yes; switching is recommended under specific circumstances—e.g. stable, well-supervised patients, clinical monitoring, provision of patient information—as biosimilars are not considered interchangeable with reference medicines	Currently none	https://health.gov.ie/wp-content/uploads/2017/08/National-Biosimilar-Medicines-Policy-Consultation-Paper-2017.pdf
				A national biosimilar policy is under development (based on a consultation paper August 2017)	
IT	Yes	Mandatory	Yes; the decision to switch rests with the physician, but biosimilar prescribing and switching is recommended and there are prescribing quotas in some regions	Position paper of the national Medicines Agency (2018 version)	http://www.aifa.gov.it/sites/default/files/pp_biosimilari_27.03.2018.pdf
NL	Yes	Voluntary	Yes; switching is recommended	Position papers of regulatory agency and Medical Profession published on their websites	https://www.cbg-meb.nl/onderwerpen/medicijninformatie-originele-biologische-medicijnen-en-biosimilars/extra-medische-informatie-voor-zorgverleners
					https://www.demedischspecialist.nl/sites/default/files/Standpunt%20Biosimilars%20Federatie%20Medisch%20Specialisten.PDF
NO	Yes	Voluntary	Yes; switching is recommended	Position paper of regulatory agency NOMA published on their website	https://legemiddelverket.no/nyheter/switching-between-a-reference-product-and-a-biosimilar
PT	Yes	Mandatory	Yes; recommendation to opt for substances which have biosimilar and prescribe and start naive patients on the most economical alternative; switching is possible under specific circumstances (e.g. pharmacovigilance)	Guidelines of the national Pharmaceutical Commission published on the website of medicines agency INFARMED	http://www.infarmed.pt/documents/15786/1816213/1_Orienta%C3%A7%C3%B5es_CNFT_Completa_Final.pdf/bd4475fc-147b-4254-a546-03b8cd63efff
					http://www.infarmed.pt/documents/15786/1816213/1_Orienta%C3%A7%C3%B5es_CNFT_Resumo_Final.pdf/a0e7f259-ec02-45a4-8700-994e712a4f14
SE	No	Not allowed	Yes; choice between reference medicine or biosimilar(s) rests with the physician. Physicians are urged to consider all factors including safety, effectiveness, and price difference. Multiple switching is not recommended	Report of the Medicines Agency with recommendation	https://www.lakemedelsverket.se/sv/tillstand-godkannande-och-kontroll/tillverkningstillstand/biologiska-lakemedel#hmainbody1
SK	Yes	Mandatory	Yes; switching is possible	Background papers explaining biosimilars (primarily addressed to patients)	https://www.sukl.sk/buxus/docs/odpovede_na_otazky_o_biologickych_liekoch.SUKL.pdf
UK	(Yes)	Generally voluntary, but not allowed for biologics	Yes; choice between reference medicine or biosimilar(s) rests with the physician. Physicians are urged to choose “best value.” Switching is allowed under certain preconditions (shared decision-making with patients, monitoring mechanisms)	Guidance document on biosimilars by regulatory agency	https://www.england.nhs.uk/medicines/biosimilar-medicines

INN = International Non-Proprietary Name.

a Bioidenticals are defined as medicines which do not differ in chemical precursors and manufacturing process. They were produced in the same production site and are marketed by different pharmaceutical companies under different brand names (co-marketing).

In all the countries studied here, physicians are expected to prescribe rationally, and retain final decision-making power on therapeutic choices. Prescribing quotas are another measure used by a number of countries to steer the use of lower-priced medicines in the off-patent sector. These are predefined targets for the share of generics or biosimilars physicians are expected to observe when prescribing. In Belgium, quotas for “cheap” medicines vary by physician specialty (from 38% for endocrinologists to 91% for dentists, with 60% for general practitioners). So-called cheap medicines include generics, biosimilars, and all originators and reference medicines with prices as low as those of generics or biosimilars (INAMI, 2020). Until April 2019 the requirement to prescribe economically was restricted to the outpatient sector; it was then extended to the inpatient sector, when medicines are dispensed by the hospital pharmacy to outpatients. In France, a performance-based component was added to capitation payments to physicians some years ago, the “rémunération sur objectifs de santé publique” (ROSP). The ROSP is based, among others, on indicators regarding generic—and since its 2016 iteration, biosimilar—prescribing. The ROSP 2016 includes a 20% biosimilar prescribing quota for insulin glargine, compared to generics quotas ranging from 62% (asthma) to 92% (statins) (Ministère des Affaires Sociales et de la Sante, 2020). Social security in France evaluates this mechanism positively, noting that the biosimilar share of insulin glargine prescriptions rose by 6.3 percentage points between 2017 and 2018 and another 5.2 percentage points between 2018 and 2019 (AMELI, 2019; AMELI, 2020). In the French inpatient setting, a similar mechanism is included in the regional quality improvement contracts (contrats d’amélioration de la qualité et de l’efficience des soins, CAQUES), some of which also consider biosimilar prescribing targets. In Germany, regional physician associations agree on biosimilar prescribing quotas with the state-level associations of payers (sickness funds); these quotas vary considerably between federal states. The biologicals most commonly subject to regional prescription targets are epoetins, infliximab, and etanercept, as well as oncologic biosimilars for rituximab and trastuzumab. These regional quotas are complemented by “integrated care contracts” between individual sickness funds and individual providers, their networks or professional bodies (so-called selective contracts). Patients enrolled in such contracts have to use participating providers, who are in turn expected to prescribe biosimilars as a preferred option. Rheumatologists working under the 2018 specialist contract with the “Techniker Krankenkasse,” one of Germany’s largest sickness funds, have a 60% biosimilar prescribing target for etanercept and 80% for infliximab and rituximab infusions (Luley and Pieloth, 2018).

Biosimilars can be prescribed to treatment-naïve patients, or a physician can change a patient’s treatment regimen from a reference medicine to a biosimilar or from one biosimilar to another (so-called switch). While all countries stress that final decision-making power rests with the prescribing clinician, switching is in general recommended, or at least not prohibited. Switching recommendations are largely bound to certain preconditions, such as shared decision-making and close monitoring. Most countries have published related guidelines, developed by the competent authorities and/or the medical profession (see Table 4).

##### Pharmacists: Substitution and Financial Incentives

One of the most important measures for the increased use of economical medicines is substitution at the pharmacy level. Generic substitution has become standard and is employed in all the studied countries except Austria and the United Kingdom (Table 5). In contrast, substitution for biologicals (i.e. dispensing of a biosimilar instead of the reference medicine or another biosimilar) is only rarely applied, and is not allowed even in countries with mandatory generic substitution. For instance, Spain has regulated by law which medicines are excluded from substitution unless the prescribing physician has explicitly allowed it; these include biologicals (Ministerio de Sanidad y Consumo, 2020). Similarly, the Slovak legislation does not include any biosimilars in the list of medicines subject to mandatory substitution.

**TABLE 5:** Measures to endorse the use of biosimilar medicines at community pharmacy.

Country	Biosimilar substitution		Generic susbstitution	Financial incentives to dispense biosimilars
	Applied	Bindingness	Bindingness	
AT	No	Not allowed	Not allowed	No financial incentives
BE	No	Not allowed	Yes, voluntary in general (mandatory for antibiotics and antimycotics)	No financial incentives
CZ	Yes	Not explicitly prohibited, but not recommended by physicians and pharmacists	Yes, voluntary	N.A.
DE	No	As of 2022 substitution takes place automatically provided that the Federal Joint Committee has determined interchangeability	Yes, mandatory	No financial incentives
DK	No	Not allowed	Yes, mandatory	No financial incentives
ES	No	Not allowed	Yes, mandatory	No financial incentives
FI	No	Not allowed	Yes, mandatory	No financial incentives
FR	No	Not allowed (legal mandate as of 2014 to implement biosimilar substitution) was abolished in the 2020 Social Insurance law	Yes, voluntary	Yes, as part of the pharmacy mark-up regulation
IE	No	Not allowed	Yes, voluntary	No financial incentives
IT	No	Not allowed	Yes, mandatory	No financial incentives (higher wholesale and pharmacy margins for generics than for originators and biosimilars)
NL	No	Not allowed	Yes, voluntary	No financial incentives
NO	No	Not allowed^a^	Yes, voluntary	No information on financial incentives
PT	No	Not allowed	Yes, mandatory (with exceptions defined in law)	No financial incentives
SE	No	Not allowed	Yes, mandatory	No financial incentives
SK	No	Not allowed^b^	Yes, mandatory	N.A.
UK	No	Not allowed	Not allowed	No financial incentives

N.A. = no information available.

a As of July 2020, a public consultation of the Ministry of Health and Social Affairs to possibly introduce biosimilar substitution is ongoing.

b Substitution regulation does not differ between generics and biosimilars. However, the statutory list of active substances, which are subject to mandatory substitution, does not include any biological.

As of 2020, biosimilar substitution is only permitted in the Czech Republic (though not recommended by practitioners). In Germany, a law passed in 2019 foresees the automatic substitution of biosimilars in pharmacies beginning in 2022, provided the Federal Joint Committee (highest decision-making body of the self-governance of health insurers and providers) has determined the interchangeability of the medicines in question and the prescribing physician has not explicitly excluded it. In France, the regulatory framework permitted the introduction of biosimilar substitution from 2014 on, under the condition that an implementing order detailing specific provisions would be passed by administrative courts, setting out the necessary requirements for building biosimilar groups and entering the biosimilars registry. However, this order has not been issued over the years, thus hindering actual implementation biosimilar substitution, and the Social Security Law of 2020 abolished biosimilar substitution completely (Ordre National des Pharmacien, 2020).

Financial incentives for dispensing biosimilars are not present in the sample of countries, with the exception of France, where pharmacy margins for biosimilars (as well as for generics not included in the internal reference price system) are calculated based on the price of the reference medicine so as to not disadvantage pharmacists who dispense lower-priced interchangeable medicines.

### Impact of Selected Measures on Pharmaceutical Expenditure Under Different Scenarios

German statutory expenditure for the six included active substances (at ex-factory price level, considering the mandatory manufacturer discount; price data as of December 2018, consumption data of 2018 for the outpatient setting only), or the “baseline scenario” for this study, amounted to € 2,223.9 million in 2018. The largest shares were 36.5% for adalimumab, 20.6% for etanercept, and 15.3% for trastuzumab.

Table 6 shows the results of the different scenarios (the baseline and hypothetical scenarios as described in Table 1) per product as well as in total, reflecting the expenditure of German public payers (sickness funds) for 2018 as well as possible savings as a share of the baseline value. In all the scenarios, the assumed measures led to savings compared to baseline. In scenario 1 (use of lowest price in the country sample), savings amounted to € 1,238.4 million (i.e. a 55.7% reduction from baseline). € 107.2 million (4.8%) would have been saved under scenario 2 (introducing a 30% price link for biosimilars), and € 347.9 million (15.6%) in scenario 3 (30% price link for biosimilars and 15% price link for reference medicines). Scenario 4 (reference price system with German prices) would lead to savings of € 401.5 million (18.1%) and scenario 5 (reference price system with all prices) to savings of € 967.3 million (43.5%) in 2018.

**TABLE 6:** Overview of potential expenditure and savings (in %) for 2018 based on the five scenarios, broken down by product.

Medicines	Baseline	Scenario 1: European prices		Scenario 2: Price link—biosimilars		Scenario 3: Price link—all		Scenario 4: RPS—DE		Scenario 5: RPS—Europe	
	Expenditure in. mio. €	Expenditure in. mio. €	Change in %	Expenditure in. mio. €	Change in %	Expenditure in. mio. €	Change in %	Expenditure in. mio. €	Change in %	Expenditure in. mio. €	Change in %
Adalimumab, 20 mg, pre-filled syringe	1.3	0.5	−62.8	1.3	0.0	1.1	−14.9	1.0	−25.9	0.6	−50.5
Adalimumab, 40 mg, pre-filled syringe	507.7	200.0	−60.6	506.9	−0.2	432.2	−14.9	320.6	−36.9	244.8	−51.8
Adalimumab, 40 mg, pre-filled pen	288.9	113.8	−60.6	288.5	−0.1	245.9	−14.9	182.1	−37.0	138.0	−52.2
Adalimumab 40 mg, vial	3.1	1.1	−62.8	3.1	0.0	2.6	−15.0	3.1	0.0	1.6	−48.8
Adalimumab 80 mg, pre-filled syringe	9.5	3.6	−62.3	9.1	−3.9	7.7	−18.3	9.2	−2.6	5.6	−41.4
Adalimumab, 80 mg, pre-filled pen	3.8	1.4	−62.8	3.8	0.0	3.2	−15.0	3.8	0.0	2.2	−43.2
Etanercept, 10 mg, pre-filled pen	0.6	0.3	−54.9	0.6	0.0	0.5	−15.0	0.6	0.0	0.3	−50.1
Etanercept, 25 mg, pre-filled syringe	1.5	0.7	−55.9	1.5	0.0	1.3	−15.0	1.5	0.0	0.7	−52.8
Etanercept, 25 mg, pre-filled pen	30.4	13.3	−56.2	30.0	−1.3	25.9	−14.8	24.7	−18.7	14.3	−53.1
Etanercerpt, 25 mg, vial	0.1	0.0	−55.9	0.1	0.0	0.1	−15.0	0.1	0.0	0.0	−51.7
Etanercept, 50 mg, pre-filled syringe	260.7	113.0	−56.7	243.0	−6.8	224.0	−14.1	234.5	−10.1	132.0	−49.4
Etanercept, 50 mg, pre-filled pen	164.7	71.6	−56.5	154.4	−6.3	141.4	−14.2	146.8	−10.9	82.7	−49.8
Infliximab, 100 mg, vial	306.4	129.3	−57.8	273.6	−10.7	254.8	−16.9	298.1	−2.7	210.3	−31.4
Pegfilgrastim, 6mg, pre-filled syringe	77.0	36.1	−53.2	77.0	0.0	65.5	−14.9	57.0	−25.9	39.1	−49.2
Rituximab, 100 mg, vial	12.2	6.3	−48.5	11.1	−9.2	10.2	−17.0	11.4	−6.7	7.9	−35.6
Rituximab, 500 mg, vial	11.0	6.5	−41.3	11.0	0.0	9.4	−15.0	11.0	0.0	7.6	−31.1
Rituximab, 1.400 mg, vial	207.1	108.6	−47.5	182.8	−11.7	170.1	−17.9	196.5	−5.1	136.6	−34.1
Trastuzumab, 150 mg, vial	279.0	145.0	−48.0	264.5	−5.2	231.8	−16.9	261.5	−6.3	191.7	−31.3
Trastuzumab 440 mg, vial	17.4	9.7	−44.5	13.1	−24.7	13.1	−24.7	17.4	0.0	11.9	−31.7
Trastuzumab 600 mg, vial	41.4	24.8	−40.2	41.4	0.0	35.2	−15.0	41.4	0.0	29.0	−30.0
Total	2,223.9	985.5	−55.7	2,116.8	−4.8	1,876.0	−15.6	1,822.4	−18.1	1,256.6	−43.5
Savings (in mio. €) compared to baseline		1,238.4		107.1		347.9		401.5		967.3	

Scen. = scenario.

Note: Sums may deviate from totals due to rounding differences.

The largest savings were achieved under scenario 1 (“European prices”), which considered official list prices of other countries within the sample. Savings in scenario 2 (“price link–biosimilars”), which assumed a 30% lower price for biosimilars compared to the reference biological, were the smallest among the five scenarios (4.8% change). A price link policy affecting both biosimilars and reference medicines upon biosimilar market entry (scenario 3: “price link–all”) would triple these savings. Scenario 4 (“RPS–DE”) led to savings of € 401.5 million by assuming the inclusion of biosimilars in the clusters of the reference price system; also considering prices in other countries (scenario 5: “RPS–Europe”) for the calculation of the reference price more than doubled these savings. This scenario achieved the second largest savings compared to baseline.

## Discussion

The aim of this contribution has been to contextualize different mechanisms for pricing and encouraging the use of biosimilars in European countries, and explore their implications for pharmaceutical expenditure. Previous studies have highlighted the dormant potential for savings from the use of biosimilars (Haustein et al., 2012; Farfan-Portet et al., 2014; Mestre-Ferrandiz et al., 2016; Troein et al., 2019; Kim et al., 2020) and have attributed it to limited competition, small price differences between reference medicines and biosimilars, and a slow market penetration due to uncertainties about safety and effectiveness among physicians and patients alike.

Earlier reviews (Kawalec et al., 2017; Moorkens et al., 2017; Vogler et al., 2019) of policies to shape the biologicals market and make use of the efficiency gains of biosimilar medicines usually focused on a smaller group of countries, and describe a situation of a few years ago. Given the emergence of new biosimilar medicines on the market, policies in this area have been changing. This work provides a more comprehensive, updated overview, which can be beneficial as more countries consider introducing policies to increase the uptake of biosimilar medicines.

Despite the fact that price link policies seem to have some potential for savings (as demonstrated by scenario 2), not all the countries studied here apply them. A reduction in the reference medicine price at the market entry of the first biosimilar would have a more substantial effect on the total savings (as shown in scenario 3), but this variant of the price link policy is less frequently used. A design of the price link policy with a price reduction of 30% for the first biosimilar in comparison with the reference medicine price and a 15% reduction in the price of reference medicine itself at the first biosimilar’s market entry seems plausible, as this approach was identified in the legislation of some of the studied countries (Table 2). However, countries that do not apply price link policies at all (such as Germany) are less likely to enforce them for biosimilars than countries where such policies are already in place for generics.

As far as tendering is concerned, the policy is employed rather rarely in the outpatient setting, while it is prevalent in the inpatient setting, documented for every country in the sample. This policy, generally known for its savings potential (Mack, 2015; WHO, 2016), can thus have considerable impact in the area of biologicals, as these are commonly used in hospitals. For instance, in November 2018, the Danish procurement agency AMGROS serving all public hospitals completed its then largest-ever tendering procedure for biologicals, including adalimumab, and expected savings of approximately € 56 million for the year to come (AMGROS, 2018). Similarly, the inclusion of biosimilars in the preference price policy schemes of the Dutch health insurers (i.e. tendering for outpatient medicines) can be assumed to have an effect on their expenditure; the preference price policy itself was deemed so successful before the inclusion of biosimilars in 2016 that other cost-containment measures like price freezes were abandoned (Panteli et al., 2016). It is difficult to anticipate if similar large price reductions as in Denmark, the Netherlands, and Norway could be achieved for tendered biosimilar medicines in Germany. Should it be possible, the savings potential could be substantial, as also scenario 1 (application of prices as in other countries) suggests. However, these instruments also have their limitations, for instance regarding the need to adjust treatment regimens for patients and possibly introducing vulnerabilities to shortages. Conducting tenders thus requires an appropriate strategy, as highlighted under the concept of “strategic procurement” promoted by the World Health Organization (WHO, 2016).

A number of countries have included biosimilars in their reference price system, and the results in scenarios 4 and 5 confirmed the savings potential of this measure. It should be noted that a reference price system requires sound competition among a sufficient number of manufacturers to realize its cost-containment potential. In Germany, in 2018, only three active substances (infliximab, erythropoetin, filgrastim) had been included in the reference price system (baseline scenario). A higher number of biosimilar suppliers on the market for a given biologic was shown to lead to lower prices of all the medicines of a specific substance (reference medicine and biosimilars) (Curto et al., 2014). Consequently, as long as reimbursement amounts for the reference groups are set based on the prices of the medicines in the cluster, lower biosimilar prices would result in lower reimbursement amounts and thus lower public pharmaceutical expenditure. In addition to the number of biological medicines grouped in a cluster, the time after patent expiry also impacts the savings potentials. As shown in scenario 4, possible savings for infliximab, which has had biosimilars on the market for more than five years, would amount to 3% compared to 37% for adalimumab, for which the general price level experienced reductions already three months after market entry of the first biosimilar in September 2018. Furthermore, the larger saving potentials in scenario 5 compared to scenario 4 can be attributed to the lower list prices in other study countries, as is also supported by scenario 1. The cost-containment potential of a reference price system has been recognized even in countries without an established reference price system, as is attested by the introduction of country-wide reimbursement amounts for adalimumab in England in April 2019 (NHS England, 2019).

Demand-side measures mainly aim to increase the market penetration of biosimilars. This can also bolster the attractiveness of the biosimilar market and lead to an increase in the number of suppliers and the effects of competition, thus enabling lower prices and reduced expenditure. All the countries leave the final decision on whether to prescribe a biosimilar to physicians. The majority of countries allow, or even recommend, switching patients from reference medicines to biosimilars, usually tied to certain preconditions like shared decision-making with patients and close monitoring. A few years ago even countries that endorsed biosimilar prescribing for naïve patients did not recommend switching for patients already on biologicals, but this has changed. This development reflects scientific insights driven by the so-called switch studies, which investigated the consequences of switching a patient’s medication to a (different) biosimilar. They found no negative impact of switching on safety and effectiveness, but highlighted the need for information and monitoring [e.g. Jørgensen et al. (2017), Belleudi et al. (2019)]. The latter is fundamentally important to enable informed choice and decision-making for patients and providers. As observed for generics over the last decade, the acceptance of biosimilar medicines by patients and health care providers, in particular prescribing doctors, plays a major role for their uptake: concerns raised so far mainly relate to safety and efficacy, while the savings potential and continuous supply are seen as advantages of biosimilars (Baji et al., 2016; Leonard et al., 2019; Kovitwanichkanont et al., 2020; Sarnola et al., 2020). These concerns need to be addressed, and trust has to be built in the quality of biosimilars, as otherwise demand-side measures would not be able to exploit their potential to foster uptake. Good communication to both patients and health professionals is key for positively impacting their attitudes (Gasteiger et al., 2020). Some countries, such as Belgium and Germany, use prescribing targets or quotas to steer the use of biosimilars, but this is not a common approach in the study sample. It is expected that such measures would contribute to cost-containment in two different ways: by directly reducing public expenditure and by encouraging biosimilar suppliers to join the market, thus strengthening competition. Generally, the implications of these measures cannot be fully evaluated at present, as their implementation is relatively recent.

In contrast to generics, substitution for biosimilars at pharmacy level is not yet commonly applied; an exception among the countries in this study is Germany, where biosimilar substitution is planned to be implemented beginning of 2022. The contribution of the measure to cost-containment will have to be evaluated in due time. Any measure that influences dispensing at pharmacy level needs to be considered in conjunction with the remuneration for community pharmacy. Any price-oriented remuneration (i.e. pharmacists are remunerated for filling prescriptions based on the price of a medicine), even if designed as a regressive margin scheme, will incentivize the dispensing of higher-priced medicines (WHO, 2020). Alternative payment schemes for community pharmacy (performance-based remuneration) have been increasingly introduced in European countries, at least as additional remuneration components (Vogler et al., 2019). In Switzerland, pharmacists are rewarded for each generic substitution (Santesuisse, 2020). Actual savings from such measures, which can possibly lead to higher payments to pharmacies, would need to be determined. In the meantime, consolidation mechanisms in price-oriented remuneration for community pharmacy, as applied in France, could be employed.

For the calculation of potential savings from the implementation of different policies for biosimilars, this work adopted the German health system perspective. The results of scenarios 1 and 5, which were based on the lowest prices among comparator countries, indicate for biologicals what previous research (Busse et al., 2017; Schneider et al., 2018) has suggested for German prices, namely that they are relatively high in the European context. While the assumption of considering prices from countries of lower income than Germany might be challenged, it should be reminded that even for high-income countries lower prices are possible, as the evidence for generic prices demonstrates (TLV, 2020). This discrepancy between biosimilars and generics prices in Germany can be attributed to the relatively recent (or still pending) implementation of policies to foster the use of biosimilars. At the same time, the other countries also do not have a long experience in the application of policy measures for biosimilars either, and would thus benefit from the cost-containment potential of biosimilars. For instance, NHS England expects annual savings of up to € 330 million until 2021 following the implementation of relevant policies (NHS England, 2020).

Each scenario that was explored for the example of Germany has shown the potential to reduce public expenditure. It can be assumed that a combination of policies to encourage the use of biosimilar medicines would have an even higher impact. The European overview of national policies presented in “Overview of Pricing and Procurement Mechanisms and Demand-Side Measures to Endorse the Use of Biosimilars in European Countries” Section of this manuscript pointed to such bundles of measures, even though policies for biosimilars have only been implemented rather recently in some countries. Some policies are complementary, whereas other measures are applied by different actors in sequence. For instance, a price is first statutorily set by the pricing authority (i.e. based on the provisions of the price regulation, e.g. a “price link”), and in the next stage a public procurer (e.g. NHS, hospital procurement agency) tenders for the biosimilar and obtains a possibly lower procurement price, or will conclude a contractual arrangement such as a managed-entry agreement.

It can be expected that the combination of supply-side and demand-side measures can amplify the effect both on the increased use of biosimilars and on savings. In particular, when in the outpatient sector tendering is combined with prescribing by INN, substitution by pharmacists and/or a reference price system, this may likely boost competition and decrease prices and, at the same time, enhance the uptake of the “winning” or “preferred” medicine, as empirical evidence has demonstrated for generics (Dylst et al., 2011; Vogler et al., 2017). While similar developments may be assumed in the biosimilar medicines market, experience is not yet available, also because of the novelty of related measures (limited implementation of biosimilar substitution in contrast to generic substitution in European countries; exclusion of biosimilars from INN prescribing in some countries, e.g. Belgium, France, and the United Kingdom; and tendering for outpatient off-patient medicines used sometimes solely for generics but not biosimilars, e.g. Sweden, if at all).

All the scenarios calculated in this manuscript led to savings for the publicly funded health system in Germany, but the scenarios based on the assumption of full implementation of a reference price system for biologicals also have an impact on private expenditure: in a reference price system (in Germany as well as in other countries), patients have to pay the difference out of pocket if they opt for medicines priced above the determined reference benchmark amount. However, the impact on patients’ expenses should be limited in extent: data only about 7.6% of all prescriptions in Germany in 2019 concerned medicines priced above the reference price (Bundesministerium für Gesundheit, 2020).

This study did not aim to provide a budget impact assessment of policy measures. Rather, by highlighting the savings potential of policies to encourage the uptake of biosimilar medicines, it adds to several budget impact assessments that have already been conducted for one or more of the biological substances investigated here (e.g. infliximab, rituximab, trastuzumab) in European countries. All those studies identified important savings potentials in the case of a change from the originator reference medicine to biosimilars (Kanters et al., 2017; Gulácsi et al., 2017; Jang et al., 2021; Brodszky et al., 2016; Brodszky et al., 2014; Jha et al., 2015; Aladul et al., 2017). While budget impact assessment is a common methodology to estimate the financial impact resulting from the use of biosimilar medicines instead of the originator biological, such an analysis requires a wealth of data (e.g. epidemiological data, volume, market research information, prices over a longer period) to build a model that complies with good practice principles for performing a budget impact assessment (Sullivan et al., 2014). This was beyond the scope of this work, which intended to provide an illustrative example of the savings potential of selected relevant biosimilars if Germany applied measures used in other countries, or changed the design of relevant measures, or had prices as those reported in other countries. This study has some limitations. Despite careful data collection on country-level policy measures (based on predefined definitions) and validation, the authors cannot exclude misunderstandings and/or errors in reporting. The same policy measures may have been implemented differently across countries, and this is a major limiting factor in the comparison. The information on policy measures in Belgium and France was not validated for mid-2020 and may reflect the situation as of the end of 2019. For the calculations of scenarios 1 and 5 (based on prices in other countries), Ireland and Portugal had to be excluded due to missing price data. Our calculations accounted for the statutory manufacturer discount set out in German legislation, but any additional commercial … discounts granted by the pharmaceutical companies could not be taken into account because of their confidential character. Price data from the other countries also reflect list prices, without consideration of confidential discounts. Furthermore, the calculations reflect a cross-sectional view based on the reality of the German pharmaceutical market in 2018; they do not consider dynamic changes (e.g. share of biosimilars, supplier’s responses on policies) over time and may not be transferrable for other systems. The illustrative calculations for Germany in “Impact of Selected Measures on Pharmaceutical Expenditure Under Different Scenarios” Section of the manuscript were conducted for some but not all the policy options presented in “Overview of Pricing and Procurement Mechanisms and Demand-Side Measures to Endorse the Use of Biosimilars in European Countries” Section. Furthermore, the descriptive comparison of policies in European countries itself is not exhaustive; rather, those policy measures that have been recognized in the literature (Kovitwanichkanont et al., 2020) as major for generic medicines were investigated here for biosimilars. Further pricing, procurement, and reimbursement policies (e.g. external price referencing; managed-entry agreements; gain-sharing, where savings are shared between the payer and the providers who have achieved them) may also be applied to generics and biosimilars, though only in few countries.

In conclusion, this article uses the international comparison as an instrument for generating evidence-based ideas for the development, implementation, or adjustment of policy measures. Specifically, it looked at a number of supply-side and demand-side measures that influence the prices and use of biosimilars, and thus their impact on expenditure, in sixteen European countries. While differences are observed among countries for individual measures, there are discernible tendencies (price link for biosimilars with few exceptions, tendering in the inpatient and occasionally outpatient setting, reference price systems, switching allowed and/or recommended, prescribing quotas in several countries, pharmacy level substitution is rather the exception than the rule). The potential for savings of some of these measures was clearly demonstrated. However, there is room for further research on the impact of other policy options (e.g. tendering or biosimilar substitution) on public expenditure not explored in this manuscript. At the same time, it is of key importance to recognize that the relative recency of biosimilars on the pharmaceutical market means that related measures have not had sufficient running time to be evaluated yet. It is therefore vital to adhere to best practice, and monitor and evaluate existing and newly implemented policies, not only regarding their effect on reducing public expenditure but more holistically, and especially in regard to patient welfare.

## Data Availability

The data analyzed in this study was obtained from the AOK Research Institute (Wissenschaftliches Institut der AOK) in Germany (consumption data of studied active substances in Germany) and from the Pharma Price Information (PPI) service (price data). The following restrictions apply: sharing of the individual data requires permission of the original data providers. Requests to access these datasets should be directed to Sabine Vogler (sabine.vogler@goeg.at).

## References

[B1] Abbott K. Shao H. Shi L. (2019). Policy Options for Addressing the High Cost of Specialty Pharmaceuticals. Glob. Health J. 3 (4), 79–83. 10.1016/j.glohj.2019.11.005

[B2] AG probiosimilars (2017). Handbuch Biosimilars. Berlin: ProGenerika e.v.

[B3] Aladul M. I. Fitzpatrick R. W. Chapman S. R. (2017). Impact of Infliximab and Etanercept Biosimilars on Biological Disease-Modifying Antirheumatic Drugs Utilisation and NHS Budget in the UK. BioDrugs 31, 533–544. 10.1007/s40259-017-0252-3 29127626

[B4] AMELI (2019). La Rosp en 2018: des résultats bien orientés après une période d’appropriation nécessaire. Available at: https://www.ameli.fr/medecin/actualites/la-rosp-en-2018-des-resultats-bien-orientes-apres-une-periode-dappropriation-necessaire (Accessed June 1, 2019).

[B5] AMELI (2020). La Rosp en 2019: des résultats en hausse pour la seconde année consécutive. Available at: https://www.ameli.fr/medecin/actualites/la-rosp-en-2019-des-resultats-en-hausse-pour-la-seconde-annee-consecutive (Accessed September 21, 2020).

[B6] AMGROS (2018). Task Force to Ensure Large Savings on Medicine. Available at: https://amgros.dk/en/knowledge-and-analyses/articles/task-force-to-ensure-large-savings-on-medicine/(Accessed September 27, 2020).

[B7] Babar Z-U-D. Ramzan S. El-Dahiyat F. Tachmazidis I. Adebisi A. Hasan S. S. (2019). The Availability, Pricing and Affordability of Essential Diabetes Medicines in 17 Low-, Middle-And High-Income Countries. Front. Pharmacol. 10, 1375. 10.3389/fphar.2019.01375 31824316 PMC6880243

[B8] Baji P. Gulácsi L. Lovász B. D. Golovics P. A. Brodszky V. Péntek M. (2016). Treatment Preferences of Originator versus Biosimilar Drugs in Crohn's Disease; Discrete Choice experiment Among Gastroenterologists. Scand. J. Gastroenterol. 51, 22–27. 10.3109/00365521.2015.1054422 26059967

[B9] Belleudi V. Trotta F. Trotta F. Addis A. Ingrasciotta Y. Ientile V. (2019). Effectiveness and Safety of Switching Originator and Biosimilar Epoetins in Patients with Chronic Kidney Disease in a Large-Scale Italian Cohort Study. Drug Saf. 42 (12), 1437–1447. 10.1007/s40264-019-00845-y 31228010 PMC6858470

[B10] Brodszky V. Baji P. Balogh O. Péntek M. (2014). Budget Impact Analysis of Biosimilar Infliximab (CT-P13) for the Treatment of Rheumatoid Arthritis in Six Central and Eastern European Countries. Eur. J. Health Econ. 15 (Suppl. 1), S65–S71. 10.1007/s10198-014-0595-3 24832837 PMC4046087

[B11] Brodszky V. Rencz F. Péntek M. Baji P. Lakatos P. L. Gulácsi L. (2016). A Budget Impact Model for Biosimilar Infliximab in Crohn's Disease in Bulgaria, the Czech Republic, Hungary, Poland, Romania, and Slovakia. Expert Rev. Pharmacoecon Outcomes Res. 16, 119–125. 10.1586/14737167.2015.1067142 26162458

[B12] Bundesministerium für Gesundheit (2020). Zuzahlung and Erstattung von Arzneimitteln. Available at: https://www.bundesgesundheitsministerium.de/zuzahlung-und-erstattung-arzneimittel.html (Accessed September 27, 2020).

[B13] Busse R. Panteli D. Schröder H. Schröder M. Telschow C. Weiss J. (2017). “Europäischer Preisvergleich für patentgeschützte Arzneimittel,” in Arzneiverordnungs-Report 2017: Aktuelle Daten, Kosten, Trends and Kommentare. Editors Schwabe U. Paffrath D. Ludwig W-D. Klauber J. (Berlin, Heidelberg: Springer), 195–208. 10.1007/978-3-662-54630-7_7

[B14] Curto S. Ghislandi S. van de Vooren K. Duranti S. (2014). Regional Tenders on Biosimilars in Italy: an Empirical Analysis of Awarded Prices. Health Policy 116 (2-3), 182–187. 10.1016/j.healthpol.2014.02.011 24602376

[B15] de Bruijn W. Ibáñez C. Frisk P. Bak Pedersen H. Alkan A. Vella Bonanno P. (2016). Introduction and Utilization of High Priced HCV Medicines across Europe; Implications for the Future. Front. Pharmacol. 7, 197. 10.3389/fphar.2016.00197 27516740 PMC4964878

[B16] Dylst P. Vulto A. Simoens S. (2011). Tendering for Outpatient Prescription Pharmaceuticals: what Can Be Learned from Current Practices in Europe? Health Policy 101 (2), 146–152. 10.1016/j.healthpol.2011.03.004 21511353

[B17] European Medicines Agency (2020a). Bisimilar Medicines: Overview. Available at: https://www.ema.europa.eu/en/human-regulatory/overview/biosimilar-medicines-overview (Accessed September 21, 2020).

[B18] European Medicines Agency (2020b). Centrally Authorised Biosimilar Medicines. Available at: https://www.ema.europa.eu/en/medicines/field_ema_web_categories%253Aname_field/Human/ema_group_types/ema_medicine/field_ema_med_status/authorised- 36/ema_medicine_types/field_ema_med_biosimilar/search_api_aggregation_ema_medicine_types/fi eld_ema_med_biosimilar (Accessed September 21, 2020).

[B19] Farfan-Portet M.-I. Gerkens S. Lepage-Nefkens I. Vinck I. Hulstaert F. (2014). Are Biosimilars the Next Tool to Guarantee Cost-Containment for Pharmaceutical Expenditures? Eur. J. Health Econ. 15 (3), 223–228. 10.1007/s10198-013-0538-4 24271016 PMC3950601

[B20] Ferrario A. Dedet G. Humbert T. Vogler S. Suleman F. Pedersen H. B. (2020). Strategies to Achieve Fairer Prices for Generic and Biosimilar Medicines. BMJ 368, l5444. 10.1136/bmj.l5444 31932323

[B23] G-BA (2017). Arzneimittel-Richtlinie/Anlage IX: Infliximab, Gruppe 1, in Stufe 1. Available at: https://www.g-ba.de/beschluesse/3132/(Accessed September 21, 2020).

[B21] GABi Online (2015). Huge Discount on Biosimilar Infliximab in Norway. Available at: http://www.gabionline.net/Biosimilars/General/Huge-discount-on-biosimilar-infliximab-in- Norway (Accessed September 21, 2020).

[B22] Gasteiger C. Jones A. S. K. Kleinstäuber M. Lobo M. Horne R. Dalbeth N. (2020). Effects of Message Framing on Patients' Perceptions and Willingness to Change to a Biosimilar in a Hypothetical Drug Switch. Arthritis Care Res. 72, 1323–1330. 10.1002/acr.24012 31233269

[B24] Godman B. Bucsics A. Vella Bonanno P. Oortwijn W. Rothe C. C. Ferrario A. (2018). Barriers for Access to New Medicines: Searching for the Balance between Rising Costs and Limited Budgets. Front. Public Health 6, 328. 10.3389/fpubh.2018.00328 30568938 PMC6290038

[B25] GÖG (2020). PPRI. Available at: https://ppri.goeg.at/ppri (Accessed September 21, 2020).

[B26] Gulácsi L. Brodszky V. Baji P. Rencz F. Péntek M. (2017). The Rituximab Biosimilar CT-P10 in Rheumatology and Cancer: A Budget Impact Analysis in 28 European Countries. Adv. Ther. 34, 1128–1144. 10.1007/s12325-017-0522-y 28397080 PMC5427122

[B27] Haustein R. de Millas C. Höer A. Häussler B. (2012). Saving Money in the European Healthcare Systems with Biosimilars. Gabi J. 1 (3-4), 120–126. 10.5639/gabij.2012.0103-4.036

[B28] INAMI (2020). Prescrire « Bon Marché». Available at: https://www.inami.fgov.be/fr/professionnels/sante/medecins/soins/Pages/prescrire-bon-marche-20150101.aspx 2018 (Accessed September 21, 2020).

[B29] Jang M. Simoens S. Kwon T. (2021). Budget Impact Analysis of the Introduction of Rituximab and Trastuzumab Intravenous Biosimilars to EU-5 Markets. BioDrugs 35, 89–101. 10.1007/s40259-020-00461-8 33368051 PMC7803676

[B30] Jha A. Upton A. Dunlop W. C. N. Akehurst R. (2015). The Budget Impact of Biosimilar Infliximab (Remsima) for the Treatment of Autoimmune Diseases in Five European Countries. Adv. Ther. 32, 742–756. 10.1007/s12325-015-0233-1 26343027 PMC4569679

[B31] Jørgensen K. K. Olsen I. C. Goll G. L. Lorentzen M. Bolstad N. Haavardsholm E. A. (2017). Switching from Originator Infliximab to Biosimilar CT-P13 Compared with Maintained Treatment with Originator Infliximab (NOR-SWITCH): a 52-week, Randomised, Double-Blind, Non-inferiority Trial. Lancet 389 (10086), 2304–2316. 10.1016/S0140-6736(17)30068-5 28502609

[B32] Kanters T. A. Stevanovic J. Huys I. Vulto A. G. Simoens S. (2017). Adoption of Biosimilar Infliximab for Rheumatoid Arthritis, Ankylosing Spondylitis, and Inflammatory Bowel Diseases in the EU5: A Budget Impact Analysis Using a Delphi Panel. Front. Pharmacol. 8, 322. 10.3389/fphar.2017.00322 28620302 PMC5449469

[B33] Kawalec P. Stawowczyk E. Tesar T. Skoupa J. Turcu-Stiolica A. Dimitrova M. (2017). Pricing and Reimbursement of Biosimilars in Central and Eastern European Countries. Front. Pharmacol. 8, 288. 10.3389/fphar.2017.00288 28642700 PMC5463127

[B34] Kay J. Schoels M. M. Dörner T. Emery P. Kvien T. K. Smolen J. S. (2018). Consensus-based Recommendations for the Use of Biosimilars to Treat Rheumatological Diseases. Ann. Rheum. Dis. 77, 165–174. 10.1136/annrheumdis-2017-211937 28866648

[B35] Kim Y. Kwon H-Y. Godman B. Moorkens E. Simoens S. Bae S. (2020). Uptake of Biosimilar Infliximab in the UK, France, Japan, and Korea: Budget Savings or Market Expansion across Countries? Front. Pharmacol. 11, 970. 10.3389/fphar.2020.00970 32733238 PMC7363801

[B36] Kovitwanichkanont T. Raghunath S. Wang D. Kyi L. Pignataro S. Morton S. (2020). Who Is Afraid of Biosimilars? Openness to Biosimilars in an Australian Cohort of Patients with Rheumatoid Arthritis. Intern. Med. J. 50, 374–377. 10.1111/imj.14753 32141205

[B37] Leonard E. Wascovich M. Oskouei S. Gurz P. Carpenter D. (2019). Factors Affecting Health Care Provider Knowledge and Acceptance of Biosimilar Medicines: A Systematic Review. J. Manag. Care Spec. Pharm. 25, 102–112. 10.18553/jmcp.2019.25.1.102 30589628 PMC10397692

[B38] Luley C. Pieloth K. (2018). Biologika: Steuern Selektivverträge die Verordnung? MvF 11, 10–11. 10.24945/mvf.06.18.1866-0533.2103

[B39] Mack A. (2015). Norway, Biosimilars in Different Funding Systems. What Works? Gabi J. 4 (2), 90–92. 10.5639/gabij.2015.0402.018

[B40] Manova M. Savova A. Vasileva M. Terezova S. Kamusheva M. Grekova D. (2018). Comparative Price Analysis of Biological Products for Treatment of Rheumatoid Arthritis. Front. Pharmacol. 9, 1070. 10.3389/fphar.2018.01070 30294275 PMC6158404

[B41] Mestre-Ferrandiz J. Towse A. Berdud M. (2016). Biosimilars: How Can Payers Get Long-Term Savings? PharmacoEconomics 34, 609–616. 10.1007/s40273-015-0380-x 26792791 PMC4863918

[B42] Ministère des Affaires Sociales et de la Sante (2020). Arrêté du 20 octobre 2016 portant approbation de la convention nationale organisant les rapports entre les médecins libéraux et l'assurance maladie signée le 25 août. Available at: https://www.legifrance.gouv.fr/jorf/id/JORFTEXT000033285608/(Accessed September 21, 2020).

[B43] Ministerio de Sanidad y Consumo (2020). Orden SCO/2874/2007, de 28 de septiembre, por la que se establecen los medicamentos que constituyen excepción a la posible sustitución por el farmacéutico con arreglo al artículo 86.4 de la Ley 29/2006, de 26 de julio, de garantías y uso racional de los medicamentos y productos sanitarios. Available at: https://www.boe.es/buscar/doc.php?id=BOE-A-2007-17420 (Accessed September 21, 2020).

[B44] Moorkens E. Vulto A. G. Huys I. Dylst P. Godman B. (2017). Policies for Biosimilar Uptake in Europe: an Overview. PLoS One 12 (12), e190147. 10.1371/journal.pone.0190147 PMC5746224 29284064

[B45] NHS England (2019). Reference Prices for Adalimumab: Letter from Matthew Swindells. Available at: https://www.england.nhs.uk/publication/reference-prices-for-adalimumab-letter-from- matthew-swindells/ (Accessed September 21, 2020).

[B46] NHS England (2020). What Is a Biosimilar Medicine? Available at: https://www.england.nhs.uk/publication/what-is-a-biosimilar-medicine/ (Accessed September 21, 2020).

[B47] Ordre National des Pharmacien (2020). Biosimilaires: la loi de financement de la Sécurité sociale pour 2020 supprime le droit de substitution. 12/02/2020. Available at: http://www.ordre.pharmacien.fr/Communications/Les-actualites/Biosimilaires-la-loi-de-financement-de-la-Securite-sociale-pour-2020-supprime-le-droit-de-substitution (Accessed September 21, 2020).

[B48] Panteli D. Arickx F. Cleemput I. Dedet G. Eckhardt H. Fogarty E. (2016). Pharmaceutical Regulation in 15 European Countries Review. Health Syst. Transit. 18 (5), 1–122. 27929376

[B49] Pauwels K. Huys I. Vogler S. Casteels M. Simoens S. (2017). Managed Entry Agreements for Oncology Drugs: Lessons from the European Experience to Inform the Future. Front. Pharmacol. 8, 171. 10.3389/fphar.2017.00171 28420990 PMC5378787

[B50] PPRI (2020). Glossary. Available at: https://ppri.goeg.at/ppri-glossary (Accessed September 21, 2020).

[B51] Santesuisse (2020). Tarifstruktur-Vertrag LOA IV/1 Vom 1. Januar 2016. Available at: https://www.pharmasuisse.org/data/docs/de/4710/Tarifstruktur-Vertrag-LOA-IV-1.pdf?v=1.0 (Accessed September 21, 2020).

[B52] Sarnola K. Merikoski M. Jyrkkä J. Hämeen-Anttila K. (2020). Physicians' Perceptions of the Uptake of Biosimilars: a Systematic Review. BMJ Open 10, e034183. 10.1136/bmjopen-2019-034183 PMC7228507 32371511

[B53] Schneider P. Vogler S. Zimmermann N. Zuba M. (2018). Preisvergleich Ausgabenstarker Arzneispezialitäten 2017. Vienna: Gesundheit Österreich GmbH.

[B54] Sullivan S. D. Mauskopf J. A. Augustovski F. Jaime Caro J. Lee K. M. Minchin M. (2014). Budget Impact Analysis-Principles of Good Practice: Report of the ISPOR 2012 Budget Impact Analysis Good Practice II Task Force. Value in Health 17, 5–14. 10.1016/j.jval.2013.08.2291 24438712

[B55] TLV (2020). International price Comparison 2019. An Analysis of Swedish Pharmaceutical Prices and Volumes Relative to 19 Other European Countries. Stockholm: Dental and Pharmaceutical Benefits Agency.

[B56] Troein P. Newton M. Patel J. Scott K. (2019). The Impact of Biosimilar Competition in Europe, October 2019. London: IQVIA.

[B57] U.S. Food and Drug Administration (2020). Biosimilar Product Information. Available at: https://www.fda.gov/drugs/biosimilars/biosimilar-product-information (Accessed September 21, 2020).

[B60] Vogler S. Zimmermann N. Haasis M. A. (2019). PPRI Report 2018. Vienna: Gesundheit Österreich GmbH.

[B59] Vogler S. Gombocz M. Zimmermann N. (2017). Tendering for Off-Patent Outpatient Medicines: Lessons Learned from Experiences in Belgium, Denmark and the Netherlands. J. Pharm. Health Serv. Res. 8 (3), 147–158. 10.1111/jphs.12180

[B58] Vogler S. Leopold C. Zimmermann N. Habl C. de Joncheere K. (2014). The Pharmaceutical Pricing and Reimbursement Information (PPRI) Initiative-Experiences from Engaging with Pharmaceutical Policy Makers. Health Pol. Tech. 3 (2), 139–148. 10.1016/j.hlpt.2014.01.001

[B61] WHO (2016). Challenges and Opportunities in Improving Access to Medicines through Efficient Public Procurement in the WHO European Region. Copenhagen: World Health Organization.

[B62] WHO (2020). WHO Guideline on Country Pharmaceutical Pricing Policies. Geneva: World Health Organization. 33950613

