# Supplementary material for: Policies to Encourage the Use of Biosimilars in European Countries and Their Potential Impact on Pharmaceutical Expenditure
Source: Front Pharmacol. 2021 Jun 25;12:625296. doi: 10.3389/fphar.2021.625296 (PMC8267415; doi:10.3389/fphar.2021.625296)
Supplement: Supplementary file 1 [file DataSheet2.docx]

**[Policies to encourage the use of biosimilars in European countries and their potential impact on pharmaceutical expenditure](https://review.frontiersin.org/Document/DownloadPDF?articleId=625296&siteId=202&userId=1129480&roleId=16)**

**Supplementary Material 2: Calculations for the savings potential:**

The dataset contains the following columns:

- Country
- Time (year / month)
- Product characteristics:
  - Trade name
  - Active substance
  - Strength
  - Pack size
  - Pharmaceutical form
  - Biosimilar (dummy variable, TRUE for biosimilars, FALSE otherwise)
- Prices
  - Ex-factory price per unit (national currency unit / EUR)
  - In case of German prices: ex-factory price under consideration of the statutory manufacturer discount of 7% (gross) / 5.88% (net)

Baseline expenditure for Germany:

- Calculate the expenditure for each medicine as of 2018 volume (in the German publicly funded health system) multiplied by the December 2018 price)

**Scenario 1:**

The German price of a trade name product would be substituted by the lowest observed price of that medicine among the surveyed countries (same trade name, active substance, strength and pharmaceutical form adjusted for the pack size).

Calculation

1. Cluster the data by active substance, strength, pharmaceutical form and trade name
2. Identify the lowest price in the surveyed countries observed in December 2018
3. Calculate the expenditure as the product of the identified price and the German 2018 volume data in all groups
4. Calculate the sum of expenditure over all groups

**Scenario 2:**

A price-link mechanism for biosimilar medicines would be applied in Germany.

It is assumed that at the time of their entry into the German market, all biosimilar medicines would be subject to a 30% price cut in comparison to the price of the reference biological at this point of time, while the price of the latter would remain unchanged.

Calculation

1. Cluster the data by active substance, strength, pharmaceutical form and trade name
2. For biosimilars, calculate the new prices as 70% of the reference biological at the time of patent expiry
3. Calculate the expenditure as the product of the 2018 volume and the price achieved under the assumed price link in case that it is lower the 2018 price, otherwise the 2018 price
4. Calculate the sum of the expenditure over all groups

**Scenario 3:**

Building on scenario 3, a price-link mechanism for biosimilar medicines in Germany would also be applied in this scenario. A price cut of 15% for the reference biological at the market entry of the first biosimilar medicine in Germany is assumed, adding to the 30% price cut for the biosimilar medicines.

Calculation

1. Cluster the data by active substance, strength, pharmaceutical form and trade name
2. For biosimilars, calculate the new prices as 70% of the reference biological at the time of patent expiry
3. For biologicals, calculate the new prices as 85% of the reference biological at the time of patent expiry
4. Calculate the expenditure as the product of the 2018 volume and the price achieved under the assumed price link in case that it is lower the 2018 price, otherwise the 2018 price
5. Calculate the sum of expenditure over all groups

**Scenario 4:**

All studied biological substances would be included in a reference price system in Germany. The specifications of the existing reference pricing system in Germany (“Festbetragssystem”) are considered: Medicines of the same active substance, strength and pharmaceutical form – adjusted for the pack size - are grouped into the same cluster, and the reference price (“Festbetrag”) per cluster is calculated based on the highest price of the lower third of medicines in the cluster.

Only German prices are used to calculate the potential savings in this scenario.

Calculation

1. Filter the data from Germany
2. Cluster the data by active substance, strength and pharmaceutical form
3. Calculate the reference price as the 1/3 quantile of unit prices
4. Calculate the expenditure as the product of 2018 volume and the price achieved under the assumed price link in case that it is lower the 2018 price, otherwise the 2018 price
5. Calculate the expenditure over all groups

**Scenario 5:**

Building on scenario 4, a reference price system would be again applied in Germany in this scenario.

The calculation to determine the reference price is repeated, but in this scenario prices from all surveyed countries are considered.

Calculation

1. Cluster the data by active substance, strength and pharmaceutical form
2. Calculate the reference price as the 1/3 quantile of unit prices
3. Calculate the expenditure as the product of 2018 volume and the price achieved under the assumed price link in case that it is lower the 2018 price, otherwise the 2018 price
4. Calculate the expenditure over all groups
