# Supplementary material for: Policies to Encourage the Use of Biosimilars in European Countries and Their Potential Impact on Pharmaceutical Expenditure
Source: Front Pharmacol. 2021 Jun 25;12:625296. doi: 10.3389/fphar.2021.625296 (PMC8267415; doi:10.3389/fphar.2021.625296)
Supplement: Supplementary file 2 [file DataSheet1.docx]

**[Policies to encourage the use of biosimilars in European countries and their potential impact on pharmaceutical expenditure](https://review.frontiersin.org/Document/DownloadPDF?articleId=625296&siteId=202&userId=1129480&roleId=16)**

Supplementary Materials 1: Overview of included medicines

| **Substance** | **Strength^2^ in mg** | **Pharmaceutical form** | **Primary packaging type** | **Month/year of patent expiration^1^** |
| --- | --- | --- | --- | --- |
| Adalimumab | 20 | Injection solution | Pre-filled syringe | 11 / 2018 |
| Adalimumab | 40 | Injection solution | Pre-filled syringe | 11 / 2018 |
| Adalimumab | 40 | Injection solution | Pre-filled pen | 11 / 2018 |
| Adalimumab | 40 | Injection solution | Vial | 11 / 2018 |
| Adalimumab | 80 | Injection solution | Pre-filled syringe | 11 / 2018 |
| Adalimumab | 80 | Injection solution | Pre-filled pen | 11 / 2018 |
| Etanercept | 10 | Injection solution | Pre-filled pen | 02 / 2016 |
| Etanercept | 25 | Injection solution | Pre-filled syringe | 02 / 2016 |
| Etanercept | 25 | Injection solution | Pre-filled pen | 02 / 2016 |
| Etanercept | 25 | Powder and solvent to prepare an injection solution | Vial | 02 / 2016 |
| Etanercept | 50 | Injection solution | Pre-filled syringe | 02 / 2016 |
| Etanercept | 50 | Injection solution | Pre-filled pen | 02 / 2016 |
| Infliximab | 100 | Powder for a concentrate to prepare an infusion solution | Vial | 02 / 2015 |
| Pegfilgrastim | 6 | Injection solution | Pre-filled syringe | 10 / 2018 |
| Rituximab | 100 | Concentrate to prepare an infusion solution | Vial | 04 / 2017 |
| Rituximab | 500 | Concentrate to prepare an infusion solution | Vial | 04 / 2017 |
| Rituximab | 1.400 | Injection solution | Vial | 04 / 2017 |
| Trastuzumab | 150 | Powder for a concentrate to prepare an infusion solution | Vial | 04 / 2018 |
| Trastuzumab | 440 | Powder for a concentrate to prepare an infusion solution | Vial | 04 / 2018 |
| Trastuzumab | 600 | Injection solution | Vial | 04 / 2018 |

^1^ Refers to the active substance. Due to data exclusivities the market exclusivity of the individual products may differ.

^2^ Refers to the active substance in the usual standard unit

Source: European Medicines Agency
